# Whether the Agricultural Insurance Policy Achieves Green Income Growth—Evidence from the Implementation of China’s Total Cost Insurance Pilot Program

**DOI:** 10.3390/ijerph19020852

**Published:** 2022-01-13

**Authors:** Zhifeng Zhang, Haodong Xu, Shuangshuang Shan, Qingzhi Liu, Yuqi Lu

**Affiliations:** 1 Department of Economics, Qingdao University, Qingdao 266071, China; sasha_china@hotmail.com (Z.Z.); francisxhd@outlook.com (H.X.); 2 School of Foreign Language Education, Qingdao University, Qingdao 266071, China; 3 Department of Economics, Shandong University of Science and Technology, Taian 271019, China; 4 School of Marxism, East China University of Political Science and Law, Shanghai 201620, China; anya.lu@outlook.com

**Keywords:** total cost insurance, green income growth, environmental pollution, public health, difference-in-difference model

## Abstract

With the rise and popularization of the concept of green sustainable development, green income growth of agricultural insurance policies has attracted wide attention. Whether green income growth can be achieved has become an important criterion for measuring an agricultural insurance policy. In this context, this paper attempts to test whether the agricultural insurance policy achieves green income growth. Based on the panel data of 31 provinces (the research sample of this paper selects 31 provincial-level units (province for short) in China, including 22 provinces, 5 autonomous regions and 4 municipalities directly under the central government. Hong Kong Special Administrative Region, Macau Special Administrative Region and Taiwan Province are not included in the research sample) from 2009 to 2020 in China, this paper empirically evaluates the triple-effect of total cost insurance pilot program (TCI) on farmers’ income, environment and public health by employing a difference-in-difference model (DID). The results show that TCI increases farmers’ income, but deteriorates the environment and residents’ health without achieving green income growth. In the analysis of heterogeneity, compared with central and western regions, farmers’ income is more likely to increase in the eastern regions. However, environmental pollution is more severe, and residents’ health deteriorates more, in eastern regions. In addition, the positive effect of TCI on farmers’ income and the deterioration of residents’ health is more obvious in areas with a higher degree of damage, while the negative effect of TCI on the environment is more obvious in areas with a lower degree of damage. Furthermore, the mechanism analysis shows that TCI not only promotes the increase in farmers’ income through insurance density, but also affects the environment and residents’ health through straw burning. Therefore, the government should raise the subsidy standard for farmers to use straw-processing equipment and also to implement differentiated subsidies in regions with different levels of economic development and areas with different degrees of damage.

## 1. Introduction

Agricultural production plays an irreplaceable role in the economic and social development of a country. It is not only the cornerstone of the development of the national economy related to food security, but also affects environmental quality and residents’ health. Agricultural production is a risky process. The risk of agricultural disasters has become a major hidden danger that threatens agricultural production activities. In order to avoid the significant negative externality of agricultural disaster risks, various countries and regions have implemented agricultural insurance and premium subsidies to transfer risks. Through risk transfer, farmers are encouraged to increase agricultural input to boost agricultural productivity and promote agricultural development. However, it has been proved that the implementation of agricultural insurance subsidy policies will aggravate soil pollution, caused by the application of fertilizers and pesticides, and air pollution, caused by improper disposal of straws, etc. Environmental pollution and residents’ health may be deteriorated over years. With the rise and promotion of concepts related to green sustainable development, the issue of green income growth in agricultural insurance policies has attracted wide attention. Whether green income growth can be achieved has become an important criterion for measuring an agricultural insurance policy [1].

During the decades of development of China’s agricultural insurance policy, farmers’ demand for agricultural insurance was relatively low. Therefore, low farmers’ insurance participation has lasted for a long time. Consequently, development of the agricultural insurance market is unsatisfactory. In this background, the Ministry of Finance, the Ministry of Agriculture and Rural Affairs, and the China Banking and Insurance Regulatory Commission jointly issued the *Notice on Launching the Pilot Work of Total Cost Insurance and Income Insurance for the Three Major Food Crops*, which decided to start subsidizing TCI in 2018. This paper only analyzes the triple-effect of TCI, but does not analyze the effect of income insurance. The Chinese government has selected Inner Mongolia, Liaoning, Anhui, Shandong, Henan, and Hubei as the pilot provinces to carry out TCI of the three major crops. TCI covers major natural disasters, major plant diseases and insect pests and accidents, etc. The insured amount covers material and service costs with additional land costs and labor costs. The insurance targets are the three major grain crops, rice, wheat, and corn. The basic information of TCI is shown in Table 1.

**Figure 1:**
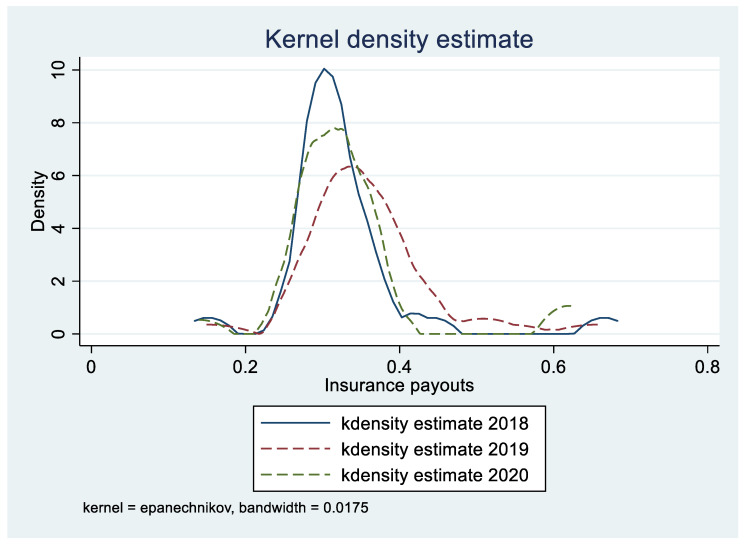
Dynamic evolution trend of the payout rate of TCI.

On the basis that the self-payment ratio of farmers is no less than 30%, China’s fiscal administration subsidizes 40% of total funding in the central, western and northeastern regions, and 35% in the east during the implementation of TCI. The pilot provinces invested a total of 827,702,200 CNY in 2020. Specifically, of which the central government fund contribution was 327,466,900 CNY, the local fiscal fund contribution was 255,250,200 CNY, and the farmers’ self-paid insurance premium amount was 244,983,100 CNY. Regarding insurance and claims settlement, the pilot provinces insured a total area of 0.9413 million hectares in 2020, an increase of 9.28% over 2019. The total insurance coverage amounted to 1,208,124,900 CNY, and the total claims amount was 79,584,600 CNY. The kernel density function graph of TCI in the pilot provinces during 2018–2020 is shown in Figure 1.

Kernel density estimation is a non-parametric estimation method of probability density function proposed by Rosenblatt [2]. This method does not make any assumptions about the specific distribution of the model, so the estimation result is more robust. Generally, the dynamic evolution process of a certain indicator’s change with the pilot year is visually presented in the form of a kernel density graph. The pilot period of this paper is from 2018 to 2020, sampling at the same interval, which is 1 year. Data for the years 2018, 2019, and 2020 are extracted, respectively, to draw a nuclear density estimation graph. Through the overall analysis of the nuclear density curve of the year evaluated, the dynamic evolution trend of the pilot insurance payout rate can be explained. As shown in Figure 1, the kernel density curve of payout rate of TCI is obviously right-tailing, and it continues to move to the right over time. At the same time, it can be seen that the degree of dispersion has continued to increase while the peak value decreases over time. The pilot insurance payout rate has shifted from a low level of aggregation to a high level of discreteness during the pilot period. The graph further shows that since the implementation of the pilot insurance policy in 2018, the protection against agricultural disasters has been enhanced. The compensation for farmers has shown a continuous increase. In light of the situation, TCI has greatly played the role of economic compensation and transfer of agricultural risks, and farmers’ income has been improved. With the rise and popularization of the concept of green sustainable development, green income growth of agricultural insurance policies has attracted wide attention. Whether green income growth can be achieved has become an important criterion for measuring an agricultural insurance policy. We need to focus on the effect of TCI on environment and public health. How TCI affect environment and health of the residents needs to be empirically tested. Whether TCI can achieve green income growth is the issue which the paper concentrates on.

In this context, this paper takes China’s TCI as an example to study the triple effect of TCI on farmers’ income, agricultural environment, and public health by employing a difference-in-difference model (DID). The study aims to find out whether TCI can achieve green income growth. The empirical research is based on the inter-provincial panel data from 31 provinces in China from 2009 to 2020. This paper not only explores the heterogeneity of policy effects, but also verifies the internal mechanism of TCI on farmers’ income, environmental pollution and public health. The empirical results can help governments around the world to pay more attention to potential environmental and health issues when optimizing agricultural insurance policies in the future. Additionally, the results help the administration to formulate agricultural insurance policies that take into account income growth, environmental protection and public health, and eventually achieve green income growth.

## 2. Literature Review

There is an intense debate among scholars about the policy effects of agricultural insurance policy on farmers’ income. Some scholars have affirmed the effects of agricultural insurance subsidy policies. For example, Babcock [3], Coble and Barnett [4] believed that subsidy policies, which increase farmers’ motivation for participating in agricultural insurance, can correct market failures. Tronstad and Bool [5] proposed that government subsidies for agricultural insurance are conducive to expanding crop planting areas and improving the efficiency of agricultural insurance. Sherrick et al. [6] found that increasing government subsidies increased the proportion of farmers participating in agricultural insurance, and that the supply of agricultural insurance will increase. A study by O’Donoghue et al. [7] showed that agricultural insurance policies have a significant role in promoting the specialized production of farmers, thereby improving the efficiency of agricultural production. However, some scholars question the positive effect of agricultural insurance policies. For example, Goodwin and Smith [8] believed that agricultural insurance subsidies have a negative impact on the insurance market price mechanism, then increase the adverse selection behaviors of farmers. That leads to the negative effect of agricultural insurance policies. Lusk [9] suggested that the governments formulate an agricultural insurance subsidy system prudently; Lusk then draws a conclusion that crop insurance subsidies will reduce farmers’ income and social welfare, based on the study from America over the past ten years. Glauber and Collins [10] found that insurance subsidies distort the structure of planting crops. The insurance makes farmers tend to plant more subsidized crops, consequently reducing market prices and offsetting the welfare effects of subsidies. Goodwin et al. [11] believed that there is no evidence that agricultural insurance subsidies can promote the growth of crop production. In addition, some scholars believe that agricultural insurance policies can bring other policy effects beside the effect on income. Yu and Wang [12] found that insurance subsidies can mitigate the disequilibrium between supply and demand in the agricultural insurance market. Feng et al. [13] found that insurance subsidy policies can adjust the demand for agricultural insurance, thereby enhancing the sustainability of farmers’ purchase of agricultural insurance. Shao et al. [14] found that agricultural insurance subsidies can promote consumption of rural residents. It is certain that the effect of implementation of agricultural insurance policy has some limitations to some extent. On the one hand, some scholars believe that the effect of agricultural insurance subsidy policy needs to be improved. For example, Zhang [15] found that farmers only receive an insurance compensation of about 0.91 CNY for every additional 1 CNY of subsidy from the government. A large amount of insurance subsidies was dissipated [16,17]. On the other hand, the implementation of agricultural insurance policies will cause damage to the environment and health [18,19].

The view that agricultural insurance policies can affect the agricultural environment and health has been confirmed by numerous studies. Some scholars believe that the agricultural insurance subsidy policy can help to reduce environmental pollution [20], and the policy will not cause serious environmental pollution [21]. Zhong [22] conducted an empirical study on farmers’ behavior of chemical use after participating in insurance. The results show that there is no significant causal relationship between insurance participation and chemical usage. However, most studies have shown that agricultural insurance policies will have an adverse effect on the environment. Some scholars believe that the use of pesticides and other chemicals will pollute the soil [23]. Agricultural insurance policies have increased environmental pollution and affected residents’ health through the use of pesticides, fertilizers and other chemicals [24]. Farmers will be more likely to obtain insurance compensation due to the fluctuation in output caused by the increase in pesticide input. The pollution problem after the implementation of the agricultural insurance subsidy policy may become more prominent without policy guidance [25]. Some scholars believe that extensive straw treatment is an important cause of agricultural pollution. Technically, the gas produced by the disposal of agricultural products such as burning straw will cause air pollution [26]. In most areas of the world, effective straw-treatment technology and equipment are not popularized. Rapid increase in the amount of straw burned has aggravated air pollution, which brings tremendous pressure to the ecological environment, thus bringing a certain degree of deterioration to the health of residents [27]. Li and Jia [28] used a fixed effect model to empirically test the impact of air pollution on the health of residents and the differences among different groups. The research sample is the balance panel data sets of the China Family Tracking Survey (CFPS) in 2016 and 2018. It was found that air pollution has a significant inhibitory effect on the health and life satisfaction of residents. Increased air pollution has brought serious health risks to residents, which vary greatly between different genders and income groups. Ren et al. [29] also reached a similar conclusion that atmospheric pollutants such as harmful gases will have a significant negative impact on the health of residents.

To sum up, the existing literature mainly focus on the analysis of the impact of agricultural insurance policies on farmers’ income. Most studies ignored the environmental pollution and public health caused by agricultural products and their disposal. There is a lack of comprehensive consideration of farmers’ income, environmental pollution, and residents’ health. In addition, the above papers are more concerned with the direct impact of agricultural insurance policies. They conducted empirical tests on the changes in food production and fertilizer use after the implementation of the policy, but they failed to clearly interpret the mechanism of the agricultural insurance policies affecting farmers’ income, environmental pollution and residents’ health. This paper attempts to make up for these shortcomings.

Compared with the existing literature, the main breakthroughs of this paper are as follows: First, there is the innovation of research perspective. This paper takes TCI as an example to test whether TCI can achieve green income growth. Considering green factors such as environmental pollution and residents’ health, which are ignored in the previous literature, this paper creatively incorporates farmers’ income, environmental pollution and residents’ health together into the triple-effect evaluation of TCI. Second, there is the innovation of model design and method selection. This paper constructs DID model, continuous DID, and triple-difference model sequentially for causal effect inference [30,31]. The study uses the DID model for benchmark regression [32], and conducts robustness tests including a parallel trend test, placebo test, replacement of explained variables and continuous DID [33]. In the heterogeneity analysis, the triple-difference interaction terms are constructed [34] to explore the heterogeneity of regions [35,36] and the heterogeneity of damage degree [37,38]. Third, an empirical test of the policy mechanism is used. This paper uses mediation effect to explore the influence mechanism of TCI. The stepwise regression coefficient test method and bootstrap test [38,39] is used to analyze the mechanism of TCI affecting farmers’ income, environmental pollution and residents’ health. As a result, this paper provides a theoretical basis for the influence mechanism of agricultural insurance policies.

## 3. Data and Methods

### 3.1. Variables Selection

In the process of agricultural production, increasing the output of agriculture and farmers’ income has always been the topic supported by the agricultural insurance policies. However, with the concept of green and sustainable agricultural production rising, environmental pollution and residents’ health are increasingly important for measuring an agricultural insurance policy. Therefore, farmers’ income is no longer used as the singular measurement indicator. Green factors such as environment and health should be taken into consideration when evaluating the performance of agricultural insurance policies. This paper considers environmental pollution and residents’ health as the green factors [34]. Taking China’s TCI as an example, the study empirically evaluates the triple effect of TCI to test whether the green income growth can be realized.

Environmental pollution caused by agricultural production is mainly soil pollution caused by excessive application of chemical fertilizers and pesticides and air pollution caused by burning straw. Most of the chemical fertilizers and pesticides used in the world’s agricultural production are of hypo-toxicity, so the harm to human health and the environment can be ignored [28]. Therefore, this paper mainly examines air pollution caused by burning straw. Residents’ health is generally measured by the incidence of major diseases issued by health institutions [29]. In order to test the triple-effect of TCI on farmers’ income, environment, and public health by using DID model, this paper selects the per capita disposable income of rural residents (Income), air pollutant emissions (Pollution) and the incidence of major diseases (Disease) as the explained variables. According to the empirical analysis in the existing literature, farmers’ income is measured by the per capita disposable income of farmers (Income) [1], the emission of air pollutants (Pollution) represents environmental pollution [25], and the health of residents is measured by the incidence of major diseases (Disease) [32]. In this paper, control variables include industrial structure (Str), per capita sown area (Sown), the degree of damage (Disaster), agricultural modernization level (Modern), urbanization level (Urban), regional average education level (Edu), economic development (Eco) and inflation rate (Inf) [14,15]. The symbol and definition of the main variables in this paper are shown in Table 2.

### 3.2. Data Source

This paper selects the panel data of 31 province-level units in China from 2009 to 2020. The statistical software Stata 15 (College Station, TX, USA) is used for empirical analysis. The data of various variables used in benchmark regression, robustness test, heterogeneity analysis and mechanism analysis come from the website of the National Bureau of Statistics of China (stats.gov.cn), China Agriculture Yearbook (cnki.net) and China Environmental Yearbook (environmentcnki.net). The descriptive statistics of each variable are shown in Table 3.

### 3.3. Research Methods

In order to analyze the triple-effect of TCI and test whether TCI can achieve green income growth, this paper uses the DID model for empirical analysis, referring to the methods of Beck et al. [30], Li et al. [31] and Gao et al. [1]. The specific difference-in-difference model is as follows:

(1) $$Y_{\mathrm{it}}=\alpha +\beta_{1}D_{\mathrm{it}}+\beta_{2}{\mathrm{control}}_{\mathrm{it}}+\lambda_{t}+\mu_{i}+\varepsilon_{\mathrm{it}}$$

where $Y_{\mathrm{it}}$ is the explained variable, including Income, Pollution or Disease. $D_{\mathrm{it}}$ is the dummy variable of the difference-in-difference policy, which is the interaction term of ${\mathrm{treat}}_{i}$ and ${\mathrm{post}}_{t}$. Inner Mongolia, Liaoning, Anhui, Shandong, Henan, and Hubei are used as the experimental group, and the value of ${\mathrm{treat}}_{i}$ is 1. The other 25 provinces are used as the control group, and the value of ${\mathrm{treat}}_{i}$ is 0. The policy implementation time point is 2018. Before 2018, the value of ${\mathrm{post}}_{t}$ is 0, and in 2018 and after, the value of ${\mathrm{post}}_{t}$ is 1. Therefore, after the implementation of TCI in the provinces of the experimental group, the value of $D_{\mathrm{it}}$ is 1, otherwise the value is 0. The coefficient $\beta_{1}$ is the core indicator to measure the effect of TCI. ${\mathrm{Control}}_{\mathrm{it}}$ represents the control variable, $\lambda_{t}$ is the year-fixed effect, $\mu_{i}$ is the province-fixed effect, and $\varepsilon_{\mathrm{it}}$ is the random error term. In the empirical analysis, in order to unify the dimensions, the logarithm of Income is processed. Income, Pollution and Disease are regressed, respectively, to the explanatory variables, in order to evaluate the triple-effect of TCI. Additionally, whether TCI achieves green income growth can be concluded.

## 4. Results

### 4.1. Benchmark Regression Analysis

Table 4 shows the results of benchmark regression analysis. Columns a, c, and e include no control variables. According to the stepwise regression method, control variables are added in the columns b, d, and f on the basis of columns a, c, and e, respectively. Columns a and b examine the causal relationship between TCI and farmer’s income. In column a, the coefficient of $D_{\mathrm{it}}$ is significant. It suggests that TCI plays an important role in promoting the per capita disposable income of farmers. The control variables are added in Column b on the basis of column a. It shows that $\beta_{1}$ is significant at the 1% significance level, which confirms the positive effect of TCI on increasing farmer’s income. Columns c and d examine the causal relationship between TCI and environmental pollution. These two columns indicate that TCI has a significant negative impact on the environment. After the control variable is added, the coefficient of the interaction term is 13.762, which is significant at the 5% significance level. The air quality in the pilot provinces during the pilot period has declined. Columns e and f test the causal relationship between TCI and residents’ health. The two columns show that TCI has increased the incidence of major diseases and has a significant negative impact on residents’ health. After the control variables is added, the coefficient of the interaction term is 0.443, which is significant at the 5% significance level. It is found in the study that TCI promotes farmers’ income but deteriorates the environment and residents’ health. Xiao and Fang [32] used the mediation effect model and the threshold model to study the impact of income gap and environmental pollution on residents’ health. It is verified that environmental pollution is an effective path for income gap to affect residents’ health. They believed that environmental pollution has a certain mediation effect on residents’ health. Meanwhile the view that environmental pollution will affect the health of residents has been confirmed in many studies [28,29]. Therefore, the deterioration of residents’ health may be caused by the aggravation of environmental pollution by TCI. It can be concluded that TCI promotes farmers’ income growth, reduces production risks, makes economic compensation and transfers risks. However, the lack of government agencies’ attention to environmental and health issues has led to the continuous deterioration of air quality and residents’ health in the pilot provinces. To sum up, green income growth has not been achieved.

### 4.2. Robustness Test

This paper uses three methods to verify the accuracy of the conclusions. The first method is parallel trend test. The second method is the placebo test. In the third method, the explained variables in Equation (1) are replaced by net operating income of farmers’ households, the number of environmental incidents, and the death rate, respectively [1,38].

#### 4.2.1. Parallel Trend Test

**Figure 2:**
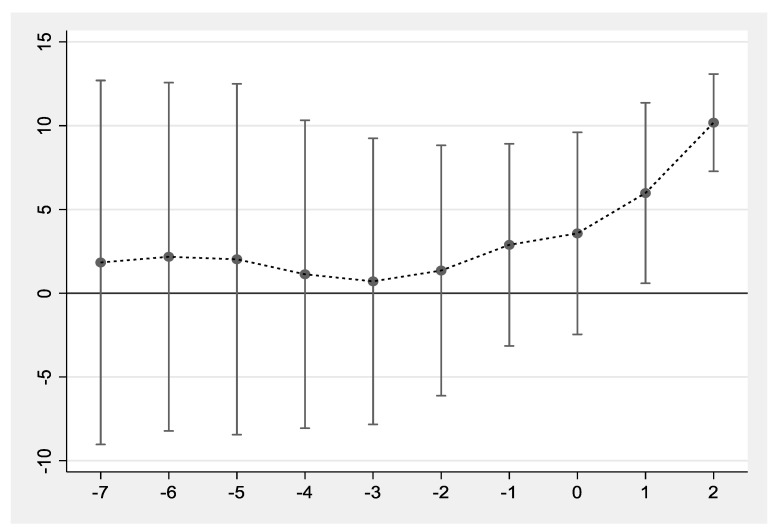
Parallel trend test.

One of the most important premises of the difference-in-difference model is to conform to the parallel trend hypothesis. The parallel trend means that the explained variables in the policy pilot provinces and the non-policy pilot provinces must have a common trend before the implementation of the policy. In this paper, referring to the test of the parallel trend hypothesis in the previous studies [30,33], the two-way fixed effect model for parallel trend test is constructed as follows.

(2) $$Y_{\mathrm{it}}=\alpha +\beta_{i}\overset{2}{\underset{k=-2}{\sum }}D^{k}{}_{\mathrm{it}}+{\tau \mathrm{control}}_{\mathrm{it}}+\lambda_{t}+\mu_{i}+\varepsilon_{\mathrm{it}}$$

where $D^{k}{}_{\mathrm{it}}$ is based on the implementation year of TCI in 2018. In 2018, the value of K is 0. For the provinces during K years before the implementation of TCI, $D^{-k}{}_{\mathrm{it}}$ is taken as 1. For provinces during K years after the implementation of TCI, the value of $D^{k}{}_{\mathrm{it}}$ is 1. In other cases, the value of $D^{k}{}_{\mathrm{it}}$ and $D^{-k}{}_{\mathrm{it}}$ are both 0. The value of K ranges from −2 to 2. The meanings of other variables are consistent with model (1). As is shown in Figure 2, before the implementation of the policy, there was no obvious trend difference in the incidence of major diseases between the pilot provinces and non-policy pilot provinces. However, the incidence of major diseases began to increase significantly after the implementation of the policy, indicating TCI has a significant impact on the health of residents. The policy effect has continued to increase within a few years after the implementation of the policy. This result fully proves that the model (1) conforms to the parallel trend assumption.

#### 4.2.2. Placebo Test

**Figure 3:**
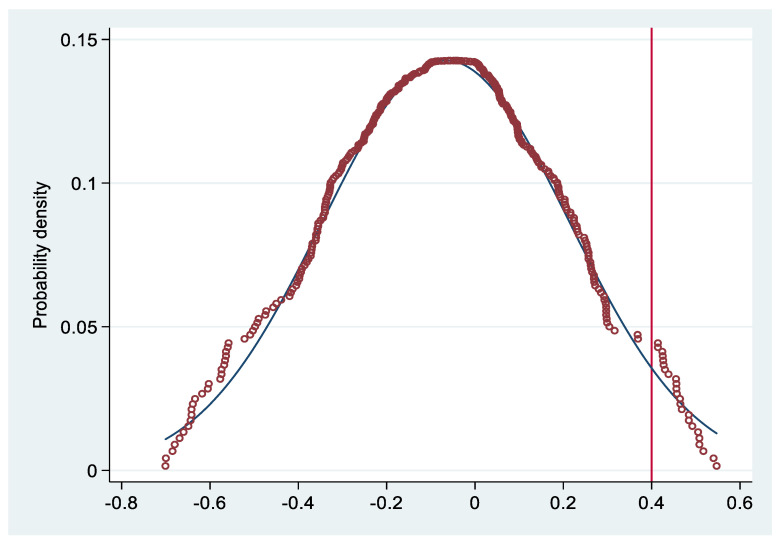
Placebo test.

In order to prove that the changes in farmers’ income, environmental pollution and residents’ health problems in the pilot provinces are indeed caused by TCI, instead of other unobservable factors, this paper conducts a placebo test referring to Lu et al. [34]. Specifically, this paper randomly selects some provinces from the 31 provinces included in the samples as the treatment group to re-estimate the model (1). The parameter estimation results of the core explanatory variables are obtained. This process is repeated 1000 times, so that 1000 coefficient estimation results can be obtained, from which the kernel density graph is drawn. The result is shown in Figure 3. The dotted line in the figure is the estimated value of the coefficient of $D^{k}{}_{\mathrm{it}}$ mentioned above. The probability density graph is of normal distribution. The coefficient estimates obtained above are obviously different from the mean value of the kernel density distribution. It is fully illustrated that the causality effect of agricultural insurance subsidy policies on farmers’ income, environmental pollution and residents’ health come from TCI, instead of other unobservable factors.

#### 4.2.3. Replace the Explained Variable

The objective of this section is to verify the accuracy of benchmark regression analysis. According to related analysis in existing studies, this section selects the net operating income of farmers’ households (Operating income), the number of environmental incidents (Environment) and the death rate (Death) as the explained variables and performed the regression test after replacing $Y_{\mathrm{it}}$ in Equation (1). We replace farmers’ per capita disposable income with the net operating income of farmers’ households [1], replaces air pollutant emissions with the number of environmental incidents [28] and the incidence of major diseases with the death rate [29]. In particular, the number of environmental incidents refers to the number of incidents that occur suddenly, cause or may cause heavy casualties and heavy property losses, or threat and damage the economic and social stability and political stability of the country in one year. Additionally, it is an important indicator to measure environmental pollution [27,28]. Table 5 shows the descriptive statistics of the surrogate variables.

Operating income is also processed by taking the logarithm in order to unify the dimension. The results of the robustness test are shown in Table 6. The results show that regardless of whether the control variables are added or not, the coefficients are all positive. The results are consistent with those of in the benchmark regression. In columns a and b, the coefficients of $D_{\mathrm{it}}$ are 0.121 and 0.169, respectively, and both are significant at the 1% significance level, which shows TCI promotes farmers’ income significantly. In columns c and d, the coefficients of $D_{\mathrm{it}}$ are 7.264 and 2.228, which is insignificant when control variables are not added. The coefficients turn out to be significant at the 10% significance level after the control variables are added. In columns e and f, the coefficients of $D_{\mathrm{it}}$ are 0.088 and 0.150. The former does not pass the significance test, while the latter is significant at the 5% level of significance. The implementation of TCI has promoted farmers’ income, but environmental pollution and residents’ health have deteriorated to some extent. The results above are basically consistent with the benchmark regression results, indicating that the results of regression are robust.

### 4.3. Continuous DID

During the implementation of TCI, the proportions of central government’s premium subsidies to the eastern, central and western regions have been different, and the ratio has increased year by year. Therefore, the intensity of policy implementation is not constant during the implementation period. In order to measure the subsidies intensity of the TCI implementation, we employ a continuous DID model in this section. According to the quasi-difference method [35], this section replaces the dummy variable that whether implemented TCI or not (Treat) with the subsidized funding of the government (Subsidy). That is to say, $D_{\mathrm{it}}$ in model (1) is replaced by the interaction term between subsidy and post (Treat × post → Subsidy × post) [38]. The estimated results of continuous DID are shown in Table 7.

The continuous DID coefficients (Subsidy×post) are positive and basically significant at the 1% significance level. It is demonstrated that, on the one hand, TCI has promoted farmers’ income growth, but the emission of air pollutants and the incidence of major diseases has increased. On the other hand, the subsidies intensity of the TCI implementation has significantly increased year by year. It is consistent with the current situation. The government makes the effort to elevate the subsidy intensity of TCI, and constantly enhance the policy effect. During the implementation period of TCI, the proportions of central government’s premium subsidies to the eastern, central and western regions have been different, and the ratio has increased year by year [38]. However, the decline of environment and public health needs to be valued. Great subsidies differences in different regions with the level of economic development also needs to be analyzed.

### 4.4. Heterogeneity Analysis

Considering the great differences in the level of economic development and the severity of natural disasters among different regions in China, this paper conducts the heterogeneous analysis of the triple-effect of TCI on farmers’ income, environment, and public health. The heterogeneity analysis of regions is based on the classification of the samples into eastern, central, and western regions. According to the different regions and levels of economic development of different provinces, China can be divided into the following three regions: eastern, central, and western regions [20,36]. In addition, agricultural production activities are particularly dependent on the environment and climate. The impact on agricultural production and the effects of agricultural insurance policies has been different since the region encountered natural disasters. Therefore, according to the different degrees of damage of the pilot provinces, this paper divides the samples into severely affected areas and less severely affected areas to analyze the heterogeneity of the degree of disaster [38].

#### 4.4.1. Heterogeneity Analysis of Regions

The heterogeneity analysis of regions is to test whether there is heterogeneity in the effects of TCI among eastern, central, and western regions in China. This paper further merges the central and western provinces referring to Yuan et al.’s division of the three major regions of China [36]. Due to the systematic differences between regions, this paper introduces the dummy variable representing differences among regions (group). For provinces in the eastern region, the value of the dummy variable is taken as 1 and other provinces taken as 0. Then, new dummy variable (D × group) is introduced into the model to construct a triple-difference model for testing [36,38]. The regression results are shown in Table 8.

The estimation results in Table 8 show that whether the control variables are added or not, the coefficients of the interaction terms (D × group) in the triple-difference model are all positive, which is basically significant at the 1% level of significance. The results show that the effects of TCI on farmers’ income, environment and public health have significant heterogeneity of regions. Specifically, the effects of TCI on farmers’ income, environmental pollution and public health in the central and western regions are significantly weaker than those in the eastern regions. In general, eastern regions in China indicate higher level of economic development, while central and western regions in China indicate lower level of economic development [18]. Compared with central and western regions, farmers’ income is more likely to increase in the regions with higher level of economic development. Environmental pollution is more severe, and residents’ health deteriorates more, in eastern regions, during the implementation of TCI. It is demonstrated that farmers’ income increases more but environmental pollution is more severe, and that residents’ health deteriorates, in the regions with high level of economic development, while the effects of TCI on farmers’ income, environment, and public health are opposite in the regions with low level of economic development.

#### 4.4.2. Heterogeneity Analysis of Degree of Disaster

The core content of the agricultural insurance policy is to ensure the stability of farmers’ income and to prevent farmers from returning to poverty after disasters. Therefore, it can be inferred that if the agricultural insurance policy achieves this objective to some extent, the policy effect in areas with severe agricultural disasters will be more obvious. This section still uses the triple-difference model to analyze the heterogeneous effect of TCI. First of all, this section refers to the classification of degree of damage in agriculture in various provinces by Liang [37]. If the mean value of the disaster-stricken area in each province over the years is greater than the overall national average, it is a severely affected area. If the mean value of the disaster-stricken area in each province over the years is equal to the national average, it is an area that is not severely affected. Specifically, this paper introduces the dummy variable (degree) representing the difference in the degree of damage. For severely-damage areas, the value of the dummy variable is taken as 1, and the value of other provinces is taken as 0. It is shown in the statistics that the overall mean value of the national degree of damage is 834.57. Therefore, for provinces where the mean value of the disaster-stricken area over the years is greater than 834.57, the dummy variable is set to 1. For provinces where the mean value of the disaster-stricken area over the years is less than 834.57, the dummy variable is set to 0. Then, new dummy variable (D × degree) is introduced into the model to construct a triple-difference model for testing [38]. The regression results are shown in Table 9.

The results in Table 9 show that the effects of TCI on farmers’ income, environmental pollution, and public health are significantly heterogeneous in areas with different degrees of damage. In columns a and b, the coefficients of the interaction terms (D × degree) are 0.651 and 0.119, respectively. The former does not pass the significance test, while the latter is significant at the 1% level of significance. It is demonstrated that compared with areas with lower degree of damage, the promoting effect of TCI on farmers’ income is more significant in areas with a higher degree of damage. In columns c and d, the coefficients are −1.221 and −0.185 but are not significant, regardless of whether the control variables are added or not. It is demonstrated that the negative effect of TCI on environmental pollution has not a significant difference between severely damaged areas and less severely damaged areas. In columns e and f, the coefficients are 0.915 and 0.098. The former is significant at the 10% level of significance, while the latter does not pass the significance test. Compared with less severely damaged areas, the negative effect of TCI on public health is more significant in severely damaged areas, after the implementation of TCI.

## 5. Mechanism Analysis

The results above clearly demonstrate the triple effects of TCI. TCI can promote farmers’ income growth with the worsening of the environment and residents’ health, which means green income growth has not been achieved. Furthermore, the mechanism that generates the result above needs to be further explored. Referring to the existing theories and literature, this section poses the following theoretical analysis beforehand: On the one hand, the implementation of TCI will increase farmers’ participation in agricultural insurance. Therefore, demand for agricultural insurance is increasing. When the supply of agricultural insurance is stable, the trading volume of agricultural insurance under the equilibrium state has increased, thereby promoting the development of agricultural insurance. The development of agricultural insurance can effectively increase agricultural production inputs by transferring agricultural business risks, optimizing agricultural risk allocation and resource allocation, thereby increasing farmers’ income [38]. On the other hand, the lack of straw-processing equipment that is environmentally friendly has worsened environmental pollution when farmers dispose of straw. In order to increase crop yields and prevent plant diseases and insect pests, farmers will burn the straw that has either been eroded by pests and diseases or has been underdeveloped. Due to the lack of environmentally friendly equipment for disposing of straw, burning straw will emit a lot of harmful gases which aggravate environmental pollution and deteriorate residents’ health [39]. Therefore, based on the theoretical analysis and academic results above, this paper proposes two hypotheses. The first is that insurance density may be a mediator for TCI to affect farmers’ income. The second is that straw burning may be a mediator for TCI to affect the environment and public health. The empirical test of the above two mechanism paths will be carried out below. This paper analyzes the internal mechanism of TCI in the framework of the difference-in-difference model by virtue of the mediation effect. The analysis aims to provide a theoretical explanation for the mechanism path that TCI affects farmers’ income, environmental pollution and public health. There are two impact paths examined here, as follows: the mechanism path of TCI affecting farmers’ income (Path a) and the mechanism path of TCI affecting the environment and health (Path b). The equation set of mediation effect model is shown in model (3).

(3) $$\{\begin{array}{c} Y_{\mathrm{it}}=cD_{\mathrm{it}}++\delta_{1}{\mathrm{control}}_{\mathrm{it}}+e_{1}\\ M_{\mathrm{it}}=aD_{\mathrm{it}}++\delta_{2}{\mathrm{control}}_{\mathrm{it}}+e_{2}\\ Y_{\mathrm{it}}=c^{\prime }D_{\mathrm{it}}+bM_{\mathrm{it}}+\delta_{3}{\mathrm{control}}_{\mathrm{it}}+e_{3} \end{array}$$

In model (3), $Y_{\mathrm{it}}$ is the explained variable, including farmers’ income, agricultural environment, and residents’ health. $M_{\mathrm{it}}$ is the mediator, including insurance density and straw burning. C represents the total effect of TCI on $Y_{\mathrm{it}}$, which is the sum of the direct effect and the indirect effect. The value $c^{\prime }$ represents the direct effect, and ab represents the indirect effect, namely the mediation effect. The descriptive statistics of the mediator are shown in Table 10.

Then, the two methods, the test of the regression coefficient by stepwise regression and the bootstrap test, are used in this part to verify the two paths. The test of the regression coefficient by stepwise regression is used in the mechanism analysis first for the path test, and then the bootstrap test is used for further verification. Whether the insurance density is the mediator of TCI affecting farmers’ income can be determined. Additionally, whether the amount of straw burning is the mediator for the causal effects of TCI on the environment and health can be determined. The mechanism of TCI on farmers’ income, agricultural environment and residents’ health can be obtained. The test results of the mediation effect are shown in Table 11 and Table 12. It can be seen from Table 11 that the impact of TCI on insurance density is 14.448. The coefficient of farmers’ income and insurance density is 0.130, which is significantly non-zero. The impact of the pilot insurance policy on the amount of straw burning is 21.584. The coefficient of the incidence of major diseases and the amount of straw burning is 0.018, which is also significantly non-zero. Therefore, the mediation effect is both established whatever the mechanism path of TCI affecting farmers’ income increase through insurance density, or the mechanism through which TCI affects the environment and health through straw burning. It is concluded on the mechanism analysis from the above. On the one hand, TCI increases insurance density, thereby increasing farmers’ income. On the other hand, TCI encourages farmers to burn straw emitting harmful gases, which will deteriorate the environment and residents’ health to some extent.

Furthermore, the bootstrap test is used to verify the results of mediation effect analysis mentioned above. The result can be seen in Table 12. This study uses a bootstrap test to sample 1000 times randomly to verify the coefficient obtained from stepwise regression, referring to the study by Wen et al. [40]. The confidence interval at 95% level of coefficient of this path a is [0.023, 0.183]. According to the principle of the bootstrap test, the confidence interval at 95% level of the path coefficient does not include 0. The existence of the mediation effect is clearly demonstrated. Similarly, the confidence interval at 95% level for measuring path b is [0.0381, 0.081]. The confidence interval at 95% level of this path does not include 0, which clearly demonstrates that the mediation effect exists and verifies the mechanism analysis mentioned above.

## 6. Discussion

From the perspectives of farmers’ income, environmental pollution and residents’ health, this paper focuses on the triple-effect evaluation on TCI and test whether TCI can achieve green income growth. The empirical study is based on inter-provincial panel data from 2009 to 2020 in China, by using difference-in-difference model. The research method used in this paper is similar to that of Gao et al. [1]. They used time-varying DID to identify the policy effect of agricultural insurance subsidy policy (AISP) from 2003 to 2012. However, this paper employs more methods, such as a continuous DID model and triple-difference model, in empirical analysis. Additionally, a continuous DID model is used to measure the subsidies intensity of the TCI implementation. The triple-difference model is used to analyze the heterogeneity characteristics of TCI, including the heterogeneity analysis of regions and heterogeneity analysis of the degree of damage. In the mechanism analysis, this paper analyzes the internal mechanism of TCI by employing the mediation effect. It is quite different from that of previous literature. Wang et al. [38] only tested the mechanism of agricultural insurance subsidy policy affecting farmers’ income by using mediation effect. Additionally, Gao et al. [1] added Mpfa and Gsa to the mechanism analysis without using mediation effect. On the basis of the above articles, the mechanism analysis aims to provide a theoretical explanation for the mechanism path that TCI affects farmers’ income, environmental pollution and public health. The mediation effect explores the following two mechanism paths: the mechanism path of TCI affecting farmers’ income (Path a) and other mechanism path of TCI affecting the environment and public health (Path b).

## 7. Conclusions

Based on the inter-provincial panel data from 31 provinces in China from 2009 to 2020, this paper takes China’s TCI as an example to study the triple-effect of TCI on farmers’ income, environment and public health. The study aims to find out whether agricultural insurance policies can achieve green income growth. The results show that TCI promotes farmers’ income, but deteriorates the environment and residents’ health, which indicates green income growth has not been achieved. Furthermore, there are significant heterogeneity characteristics in the effects of TCI on farmers’ income, environment and public health. Compared with central and western regions, farmers’ income is more likely to increase in the eastern regions. However, environmental pollution is more severe, and residents’ health deteriorates more, in eastern regions, during the implementation of TCI. In addition, the positive effect of TCI on farmers’ income and the deterioration of residents’ health is more obvious in areas with a higher degree of damage, while the negative effect of TCI on environment is more obvious in areas with a lower degree of damage. In terms of internal mechanism, on the one hand, TCI increases the insurance density, thereby increasing farmers’ income. On the other hand, TCI encourages farmers to burn straw emitting harmful gases, which is harmful to the environment and residents’ health to some extent, without achieving green income growth. The conclusions provided by empirical analysis can help governments around the world to formulate agricultural insurance policies that take environment and public health into consideration and then achieve green income growth. Based on the above empirical analysis and discussion, this paper puts forward the following recommendations about how to achieve green income growth:

First, from the perspective of environment and health, TCI has not achieved green income growth. The government should not merely focus on the increase in food production and farmers’ income during the implementation of agricultural insurance policies, they should also pay more attention to environment and public health. It is required that governments of various countries pay attention to the green income-increasing effect that involves the environment and health. In this way, the well-being of residents can be truly improved. A reasonable increase in the subsidy standards is needed for farmers to use straw-processing equipment more actively. Additionally, the subsidies should be granted to special machinery purchase to encourage environmentally friendly production and post-processing of straw and to promote the construction of environmentally friendly agricultural facilities in rural areas. As a result, environmental pollution caused by straw burning can be reduced, and the health of residents can be guaranteed.

Second, the government should implement differentiated agricultural insurance policies based on the heterogeneity of regions and degrees of disaster. To increase the level of government subsidies, the government should release policies that favor the central and western regions and areas that are not severely affected with regard to farmers’ income. In terms of reducing environmental pollution, policies that favor the central and western regions and severely affected areas should be implemented. In terms of the health of residents, the government should implement policies that favor the central and western regions and areas that are not severely affected.

Third, the government should improve the agricultural insurance subsidy policy system, continue to expand the scope of subsidies, and form a subsidy system of “bulk agricultural products plus local advantageous varieties”. Not only should administration increase premium subsidies, but also promote reinsurance subsidies, management fee subsidies, and tax incentives, so as to increase farmers’ participation in agricultural insurance. With the increase in insurance density, farmers’ income can be increased continuously. Together with the promotion of the use of straw-processing equipment and the construction of environmentally friendly agricultural facilities, green income growth can be achieved in the future.

## Figures and Tables

**Table 1:** Basic situation of TCI.

Pilot Province	Pilot Insurance	Crops Applied	Insured per Hectare	Premium per Hectare	Premium Rate
Inner Mongolia	TCI	Corn	Paddy field, 40	Paddy field, 2.4	Paddy field, 6%
			Dry land, 36.67	Dry land, 2.93	Dry land, 8%
			Paddy land, 56.67	Paddy field, 3.4	Paddy field, 6%
			Dry land, 26.67	Dry land, 3.47	Dry land, 8%
Liaoning	TCI	Corn	46.67	4.67	10%
Anhui	TCI	Rice	66.67	4	6%
Shandong	TCI	Wheat	62	2.47	3.98%
Henan	TCI	Wheat	60	3	5%
Hubei	TCI	Rice	73.33	4.4	6%

**Table 2:** Variable description.

Variable Type	Variables	Symbol	Variable Definition
Explained variables	Per capita disposable income of rural residents	Income	Income of rural households after deducting various taxes and fees paid to the state and various social insurances/the number of rural population
	Air pollutant emissions	Pollution	Emissions of harmful gases
	The incidence of major diseases	Disease	The number of people suffering from a major disease specified by the national health agency standards/total population
Explanatory variable	Interaction term of difference-in-difference	D (Treat × post)	Six provinces, Inner Mongolia, Liaoning, Anhui, Shandong, Henan, and Hubei, are the experimental group, and the remaining 25 provinces are the control group. Treat is taken as 1 in Inner Mongolia, Liaoning, Anhui, Shandong, Henan, and Hubei 6 provinces, otherwise treat = 0 The post is taken as 1 in 2018 and later, post = 0 before 2018
Control variables	Industrial structure	Str	The proportion of agricultural output value in the overall economic structure
	Per capita sown area	Sown	Rural sown area/total rural population
	Degree of damage	Disaster	The area of affected land
	Agricultural modernization level	Modern	Total power of agricultural machinery/total sown area of crops
	Urbanization rate	Urban	The proportion of urban population in total population
	Regional average education level	Edu	Regional average education level = (6 × P elementary school + 9 × P junior high school + 12 × P high school + 16 × P college or above)/(P elementary school + P junior high school + P high school + P college or above), P represents the education of each degree Population.
	Economic development	Eco	GDP per capita
	Inflation rate	Inf	Based on CPI in 2009

**Table 3:** Descriptive statistics of each variable.

Statistic	Variable	Unit	Means	Standard Deviation	Min	Max	Observations
Income	Per capita disposable income of farmers	CNY	10,665.251	5581.553	2723.8	33,195.2	279
Pollution	Air pollutant emissions	Ten thousand tons	64.403	42.595	4.06	180.11	217
Disease	Incidence of major diseases	%	5.342	2.796	−1.01	11.47	372
D	Difference-in-difference interaction term	-	0.048	0.214	0	1	372
Str	Industrial structure	%	10.485	7.723	0.3	118.6	371
Sown	Sown area per capita	Thousand hectares/person	2.714	1.643	0.31	10.06	310
Disaster	Extent of disaster	Thousand hectares	834.567	795.577	2.5	4223.7	308
Modern	Agricultural modernization level	Tons/thousand hectares	0.661	0.343	0.245	2.4626	372
Urban	Urbanization rate	%	54.793	13.789	22.6	89.6	319
Edu	Average level of education in the region	%	2.487	0.891	0.969	6.75	372
Eco	economic development	Ten thousand CNY	42,258.07	27,133	3.293	140,211.2	372
Inf	inflation rate	%	108.857	5.925	48.162	142.4	372

**Table 4:** Benchmark regression.

Variable	Income		Pollution		Disease	
	(a)	(b)	(c)	(d)	(e)	(f)
D	0.016 *	0.014 ***	17.589 **	13.762 **	0.287	0.443 **
	(0.027)	(0.019)	(6.756)	(6.339)	(0.328)	(0.360)
Str		0.097 ***		0.127 *		0.014
		(0.009)		(0.064)		(0.006)
Sown		0.238 ***		−8.218 **		−0.163 **
		(0.013)		(3.809)		(0.067)
Disaster		−0.344 **		0.003		−0.009
		(0.158)		(0.003)		(0.003)
Modern		0.089 ***		32.553 **		−0.005
		(0.031)		(12.720)		(0.230)
Urban		0.017 ***		0.773		−0.012
		(0.004)		(1.196)		(0.032)
Edu		−0.006		−6.551		0.289
		(0.043)		(13.152)		(0.321)
Eco		0.006 *		0.026		1.394 *
		(0.003)		(0.024)		(0.725)
Inf		0.524 ***		−0.117		−0.003
		(0.025)		(0.108)		(0.003)
Cons	8.462 ***	8.132 ***	77.557 ***	56.763	1.883 ***	2.738 **
	(0.011)	(0.271)	(1.612)	(56.968)	(0.083)	(1.200)
Province-fixed effect	Control	Control	Control	Control	Control	Control
Year-fixed effect	Control	Control	Control	Control	Control	Control
Observations	279	186	217	185	372	247
R-squared	0.989	0.992	0.695	0.758	0.559	0.585

Notes: The parentheses are the clustered standard errors at the Prefecture-level province level. ***, ** and * indicate significant at the 1%, 5% and 10% levels, respectively.

**Table 5:** Descriptive statistics of surrogate variables.

Statistic	Variable	Unit	Means	Standard Deviation	Min	Max	Observations
Operating income	Net operating income of farmer households	CNY	2065.789	808.7999	1110	6694	341
Environment	Number of environmental incidents	Number of times	13.67778	26.53685	1	250	270
Death	Death rate	%	2.411258	1.890643	0.1	8.92	310

**Table 6:** Robustness test.

Variable	Operating Income		Environment		Death	
	(a)	(b)	(c)	(d)	(e)	(f)
D	0.121 ***	0.169 ***	7.264	2.228 *	0.088	0.150 **
	(0.039)	(0.045)	(6.072)	(8.967)	(0.140)	(0.110)
Str		0.015		0.026		−0.163 **
		(2.027)		(0.090)		(0.067)
Sown		−0.064 **		8.399		−0.433
		(0.027)		(8.670)		(0.300)
Disaster		0.015		0.457 *		0.535
		(0.041)		(0.251)		(0.472)
Modern		0.135		3.754		−0.293
		(0.128)		(22.602)		(0.602)
Urban		−0.002		7.577		0.048
		(0.009)		(5.841)		(0.080)
Edu		0.111		−20.823		0.515
		(0.092)		(26.581)		(0.748)
Eco		0.270		0.222		0.208
		(0.268)		(0.136)		(0.244)
Inf		−0.041		0.404 ***		0.006
		(3.077)		(0.089)		(0.004)
Cons	7.185 ***	7.215 ***	16.533 ***	−401.141	1.883 ***	2.738 **
	(0.022)	(0.486)	(4.278)	(279.446)	(0.083)	(1.200)
Province-fixed effect	Control	Control	Control	Control	Control	Control
Year-fixed effect	Control	Control	Control	Control	Control	Control
Observations	341	247	270	239	310	247
R-squared	0.913	0.925	0.056	0.243	0.559	0.585

Notes: The parentheses are the clustered standard errors at the Prefecture-level province level. ***, ** and * indicate significant at the 1%, 5% and 10% levels, respectively.

**Table 7:** The results of continuous DID.

Variable	Income		Pollution		Disease	
	(a)	(b)	(c)	(d)	(e)	(f)
D	0.024	0.014	18.044 **	14.018 **	0.330	0.456
	(0.026)	(0.018)	(6.755)	(6.047)	(0.328)	(0.343)
Subsidy × post	0.715 ***	0.254 **	0.471 ***	0.215 ***	0.343 ***	0.113 ***
	(0.108)	(0.117)	(0.074)	(0.056)	(0.060)	(0.037)
Str		0.630 ***		0.195 **		0.225
		(0.039)		(0.074)		(0.174)
Sown		−0.009		−12.680 **		−0.061
		(0.014)		(4.947)		(0.058)
Disaster		−0.207 *		0.004		−5.955
		(0.113)		(0.003)		(8.134)
Modern		0.092 ***		35.336 ***		0.003
		(0.032)		(11.706)		(0.211)
Urban		0.020 ***		−0.651		0.044 *
		(0.005)		(1.125)		(0.023)
Edu		−0.013		1.212		−0.055
		(0.053)		(13.642)		(0.209)
Eco		−0.015 *		−0.614		0.240 ***
		(0.008)		(1.425)		(0.074)
Inf		0.562 ***		−0.096		−0.004 *
		(0.038)		(0.100)		(0.002)
Cons	8.451 ***	7.964 ***	77.115 ***	120.534 **	5.486 ***	0.442
	(0.010)	(0.252)	(1.621)	(52.668)	(0.133)	(2.816)
Province-fixed effect	Control	Control	Control	Control	Control	Control
Year-fixed effect	Control	Control	Control	Control	Control	Control
Observations	272	179	214	183	364	240
R-squared	0.991	0.993	0.696	0.767	0.582	0.616

Notes: The parentheses are the clustered standard errors at the prefecture-level province level. ***, ** and * indicate significant at the 1%, 5% and 10% levels, respectively.

**Table 8:** Heterogeneity Analysis of Regions.

Variable	Income		Pollution		Disease	
	(a)	(b)	(c)	(d)	(e)	(f)
D	0.016	0.014	17.589 **	13.762 **	0.287	0.443
	(0.027)	(0.019)	(6.756)	(6.339)	(0.328)	(0.360)
D × group	0.715 ***	0.254 **	0.471 ***	0.215 ***	0.343 ***	0.113 ***
	(0.108)	(0.117)	(0.074)	(0.056)	(0.060)	(0.037)
Str		−8.597 **		0.195 **		0.225
		(3.986)		(0.074)		(0.174)
Sown		−677.673 **		−12.680 **		−0.061
		(247.910)		(4.947)		(0.058)
Disaster		−0.207 *		0.004		−5.955
		(0.113)		(0.003)		(8.134)
Modern		0.089 ***		35.336 ***		0.003
		(0.031)		(11.706)		(0.211)
Urban		0.017 ***		−0.651		0.044 *
		(0.004)		(1.125)		(0.023)
Edu		−0.006		1.212		−0.055
		(0.043)		(13.642)		(0.209)
Eco		−0.015 *		−0.614		0.240 ***
		(0.008)		(1.425)		(0.074)
Inf		6.485		−0.096		−0.004 *
		(37.704)		(0.100)		(0.002)
Cons	8.462 ***	8.132 ***	77.557 ***	56.763	5.465 ***	2.320
	(0.011)	(0.271)	(1.612)	(56.968)	(0.132)	(3.247)
Province-fixed effect	Control	Control	Control	Control	Control	Control
Year-fixed effect	Control	Control	Control	Control	Control	Control
Observations	279	186	217	185	372	247
R-squared	0.989	0.992	0.695	0.758	0.582	0.616

Notes: The parentheses are the clustered standard errors at the prefecture-level province level. ***, ** and * indicate significant at the 1%, 5% and 10% levels, respectively.

**Table 9:** Heterogeneity Analysis of Damage Degree.

Variable	Income		Pollution		Disease	
	(a)	(b)	(c)	(d)	(e)	(f)
D	0.009	0.096 ***	17.080 **	13.874 **	0.307	0.371
	(0.008)	(0.032)	(7.125)	(6.466)	(0.269)	(0.435)
D × degree	0.651	0.119 ***	−1.221	−0.185	0.915 *	0.098
	(0.029)	(0.035)	(4.469)	(2.751)	(0.501)	(0.268)
Str		0.522 ***		0.177 **		0.312 ***
		(0.023)		(0.081)		(0.081)
Sown		0.583 ***		−11.137 **		−0.135 *
		(0.027)		(5.422)		(0.071)
Disaster		−0.357 **		0.763 ***		0.897 ***
		(0.149)		(0.135)		(0.158)
Modern		0.087 **		34.439 ***		−0.012
		(0.032)		(12.474)		(0.231)
Urban		0.018 ***		0.840		−0.013
		(0.004)		(1.171)		(0.032)
Edu		0.008		−5.380		0.273
		(0.042)		(13.690)		(0.320)
Eco		0.567 ***		0.570 ***		0.412 ***
		(0.100)		(0.089)		(0.146)
Inf		0.659 ***		−0.087		−2.145 ***
		(0.028)		(0.109)		(0.506)
Cons	8.462 ***	8.088 ***	77.557 ***	56.839	5.465 ***	2.374
	(0.011)	(0.247)	(1.615)	(56.811)	(0.127)	(3.247)
Province-fixed effect	Control	Control	Control	Control	Control	Control
Year-fixed effect	Control	Control	Control	Control	Control	Control
Observations	279	186	217	185	372	247
R-squared	0.989	0.993	0.695	0.758	0.568	0.585

Notes: The parentheses are the clustered standard errors at the Prefecture-level province level. ***, ** and * indicate significant at the 1%, 5% and 10% levels, respectively.

**Table 10:** Descriptive statistics of mediator.

Statistic	Variable	Unit	Means	Standard Deviation	Min	Max	Observations
ID	Insurance density	CNY per capita	4075.604	16,209.47	112.624	186,156.4	371
Burn	Straw burning	Ten thousand tons	184.353	146.513	4.6	716.09	372

**Table 11:** Stepwise test regression coefficients.

Mechanism	Path a			Path b		
Variable	Income	ID	Income	Disease	Burn	Disease
D	1.986 (0.192)	14.448 * (7.337)	0.108 (0.442)	0.443 (0.360)	21.584 ** (9.293)	0.054 (0.252)
ID	-		0.130 (0.126)	-	-	-
Burn	-	-	-	-	-	0.018 * (0.011)
Control variables	Control	Control	Control	Control	Control	Control
Province-fixed effect	Control	Control	Control	Control	Control	Control
Year-fixed effect	Control	Control	Control	Control	Control	Control

Notes: The parentheses are the clustered standard errors at the prefecture-level province level. ** and * indicate significant at the 5% and 10% levels, respectively.

**Table 12:** Bootstrap test.

Mechanism	Path a	Path b
Confidence interval	[0.023, 0.183]	[0.038, 0.081]

## Data Availability

Not applicable.

## References

[1] Gao Y., Shu Y., Cao H., Zhou S., Shi S. (2021). Fiscal policy dilemma in resolving agricultural risks: Evidence from China’s agricultural insurance pilot. Int. J. Environ. Res. Public Health.

[2] Rosenblatt M. (1956). Remarks on some nonparametric estimates of a density function. Ann. Math. Statist..

[3] Babcock B.A. (2011). Time to Revisit Crop Insurance Premium Subsidies?.

[4] Coble K.H., Barnett B.J. (2013). Why do we subsidize crop insurance?. Am. J. Agric. Econ..

[5] Tronstad R., Bool R.U.S. cotton acreage response due to subsidized crop insurance. Proceedings of the Selected Paper Prepared for Presentation at the Agricultural and Applied Economics Association, CAES&WAEA Joint Annual Meeting.

[6] Sherrick B.J. (2004). Factors influencing farmers’ crop insurance decisions. Am. J. Agric. Econ..

[7] O’Donoqhue E.J., Roberts M.J., Key N. (2009). Did the federal crop insurance reform act alter farm enterprise diversification?. J. Agric. Econ..

[8] Goodwin B.K., Smith V.H. (2013). What harm is done by subsidizing crop insurance?. Am. J. Agric. Econ..

[9] Lusk J.L. (2017). Distributional effects of crop insurance subsidies. Appl. Econ. Perspect. Policy.

[10] Glauber J.W., Colins K.J. (2002). Crop insurance, disaster assistance and the role of the federal government in providing catastrophic risk protection. Agric. Financ. Rev..

[11] Goodwin B.K., Vandeveer M.L., Deal J.L. (2004). An empirical analysis of acreage effects of participation in the federal crop insurance program. Am. J. Agric. Econ..

[12] Yu Y., Wang E. (2009). Co-integration analysis of the impact of policy subsidies on China’s agricultural insurance market. China Rural Econ..

[13] Fu J., Zhang L., Wang X. (2012). A meta-analysis of the influencing factors of agricultural insurance demand and the adjustment effect of fiscal subsidies. Macroeconomics.

[14] Shao Q., Bai L., Zhang M. (2017). The impact of agricultural insurance on the consumption and utility of rural households-and on the significance of agricultural insurance in anti-poverty. Insur. Stud..

[15] Zhang Z. (2017). Evaluation of the effect of China agricultural insurance premium subsidy funds: Methods and evidence. Financ. Res..

[16] Zhang Y., Shi H. (2007). Subsidies, welfare and policy-based agricultural insurance—An in-depth discussion based on welfare economics. J. Zhejiang Univ. Humanit. Soc. Sci. Ed..

[17] Zhao S., Wang W. (2012). Analysis of the efficiency and strategy of agricultural insurance subsidies under asymmetric information. Insur. Stud..

[18] Jiang S., Jia S., Jiang S. (2015). Analysis of China’s agricultural insurance premium subsidy efficiency and its influencing factors-based on inter-provincial panel data from 2010 to 2013. Insur. Stud..

[19] He X., Tuo G., Xie Y. (2019). Central and local responsibility sharing of agricultural insurance premium subsidies: Based on the perspective of regional equity. Insur. Stud..

[20] Fu X., Li X., Xu X., Wang Y., Liang P. (2020). Does policy-supported agricultural insurance reduce non-point source pollution? Evidence from China. Fresenius Environ. Bull..

[21] Claassen R., Langpap C., Wu J.J. (2017). Impacts of federal corp insurance on land use and environmental quality. Am. J. Agric. Econ..

[22] Zhong F., Ning M., Xing L. (2007). Does crop insurance influence agrochemical uses under current Chinese situations? A case study in the Manasi watershed, Xinjiang. Agric. Econ..

[23] Liu Y., Yue L., Li J. (2011). Evaluation of heavy metal contamination and its potential ecological risk to the soil in Taiyuan, China. Acta Sci. Circumstantiae.

[24] Cao H., Qi Y., Chen J., Shao S., Lin S. (2021). Incentive and coordination: Ecological fiscal transfers’ effects on eco-environmental quality. Environ. Impact Assess. Rev..

[25] Smith V.H., Goodwin B.K. (2013). The environmental consequences of subsidized risk management and disaster assistance programs. Annu. Rev. Resour. Econ..

[26] Yuan W., Lu N., Song J., Chen Q., Yan J., Wang W., Kang X., Wang S. (2018). Impact of burning and water corruption of crop residues on environment. Acta Agric. Zhejiangensis.

[27] Peng L., Zhang Q., He K. (2016). Emissions inventory of atmospheric pollutants from open burning of crop residues in China based on a national questionnaire. Res. Environ. Sci..

[28] Li Z., Jia C. (2021). Research on the impact of air pollution on the health level of residents. Mod. Econ. Res..

[29] Ren M., Hu M., Wang Z. (2021). Analysis of the influencing factors of Chinese residents’ health level. J. Chizhou Univ..

[30] Beck T., Levine R., Levkov A. (2010). Big bad banks: The winners and losers from bank deregulation in the United States. J. Financ..

[31] Li P., Lu Y., Wang J. (2016). Does flattening government improve economic performance? Evidence from China. J. Dev. Econ..

[32] Xiao Q., Fang S. (2021). An empirical analysis of the impact of income gap and environmental pollution on residents’ health. Stat. Decis..

[33] Wang L., Zhu L. (2019). Trade openness and fiscal expenditure cyclicality: Evidence from the PSM-DID natural experiment. Econ. Dev..

[34] Lu Y., Tao Z., Zhu L. (2017). Identifying FDI spillovers. J. Int. Econ..

[35] Nunn N., Qian N. (2011). The impact of potatoes on old world population and urbanization. Q. J. Econ..

[36] Yuan D., Zhang J., Han J. (2010). Resident Consumption, Fiscal Expenditure and Regional Effect Differences—An Empirical Analysis Based on Dynamic Panel Data Model. Stat. Res..

[37] Liang L. (2011). Comparison and Choice of the Determination Methods of China’s Food Insurance Net Rates. Quant. Econ. Tech. Econ..

[38] Wang L., Fang H., Xie F. (2020). Performance evaluation of China’s agricultural insurance subsidy policy: Empirical evidence from multiple periods of DID. J. Cent. Univ. Financ. Econ..

[39] Tu X., Yang X., Zhang J., Luan X., Ning K. (2020). Analysis of geographical characteristics of China’s straw burning fire spots from 2014 to 2019. Geogr. Res..

[40] Wen Z., Marsh H.W., Hau K.T. (2010). Structural equation models of latent interactions: An appropriate standardized solution and its scale-Free properties. Struct. Equ. Model..

